# Pulse Wave Cycle Features Analysis of Different Blood Pressure Grades in the Elderly

**DOI:** 10.1155/2018/1976041

**Published:** 2018-05-22

**Authors:** Xiao-Juan Hu, Lei Zhang, Jia-Tuo Xu, Bao-Cheng Liu, Jian-Ying Wang, Yan-Long Hong, Li-Ping Tu, Ji Cui

**Affiliations:** ^1^Shanghai Innovation Center of TCM Health Service, Shanghai University of Traditional Chinese Medicine, Shanghai 201203, China; ^2^Basic Medical College, Shanghai University of Traditional Chinese Medicine, Shanghai 201203, China

## Abstract

**Background and Objective:**

The same range of blood pressure values may reflect different vascular functions, especially in the elderly. Therefore, a single blood pressure value may not comprehensively reveal cardiovascular function. This study focused on identifying pulse wave features in the elderly that can be used to show functional differences when blood pressure values are in the same range.

**Methods:**

First, pulse data were preprocessed and pulse cycles were segmented. Second, time domain, higher-order statistics, and energy features of wavelet packet decomposition coefficients were extracted. Finally, useful pulse wave features were evaluated using a feature selection and classifier design.

**Results:**

A total of 6,075 pulse wave cycles were grouped into 3 types according to different blood pressure levels and each group was divided into 2 categories according to a history of hypertension. The classification accuracy of feature selection in the 3 groups was 97.91%, 95.24%, and 92.28%, respectively.

**Conclusion:**

Selected features could be appropriately used to analyze cardiovascular function in the elderly and can serve as the basis for research on a cardiovascular risk assessment model based on Traditional Chinese Medicine pulse diagnosis.

## 1. Introduction

Pulse diagnosis is very important in Traditional Chinese Medicine (TCM), and research has focused on obtaining objective evidence for the technique [1, 2]. Pulse waves include objective information used in TCM pulse diagnosis. The time domain features of pulse waves have physiological significance and reflect the duration and amplitude of percussion waves, tidal waves, and dicrotic waves. The frequency and time domain features can reflect the disease state [3], especially in cardiovascular disease [4]. Early research [5] found that time domain features reflect hypertension. Recent studies have sought to identify a scientific correlation between pulse patterns (wiry pulse, slippery pulse, and others) and blood pressure using a computational approach. For example, the association between pulse waves and hemodynamic parameters has been studied in hypertensive patients [6, 7], and research has shown that blood pressure values can be predicted by pulse waves [8]. Pulse waves and blood pressure values are closely associated [9–11]. According to TCM, most hypertensive patients have a wiry pulse [8]. A wiry pulse is also commonly found in normotensive elderly, especially in those over 60 years old. Moreover, the elderly are at higher risk for hypertension. In the elderly, it is unclear how to distinguish between hypertensive patients taking blood pressure medication and normotensive, using pulse waves with the same blood pressure values. In our previous studies, a series of features, including time domain (TD), energy (*E*), and higher-order statistics (HOS) features of wavelet packet decomposition coefficients (WPDC), were used in pulse classification of health versus subhealth and atherosclerosis versus nonarteriosclerosis [12]. The results have proved the feasibility of above features in pulse analysis. So we hypothesized that time domain (TD), energy (*E*), and higher-order statistics (HOS) features of wavelet packet decomposition coefficients (WPDC) in the pulse wave cycle, which can identify signal characteristics [13], may reveal differences in pulse waves within the same range of blood pressure values in hypertensive and normotensive.

This study focused on individuals over 60 years of age to identify useful features in the pulse wave cycle that can demonstrate differences between hypertensive and normotensive, within the same range of blood pressure values. In this paper, firstly, the methods were introduced including the pulse data acquisition, preprocessing and pulse wave cycle segmenting, and feature extraction. Secondly, the experiments design and result were described. Thirdly, some details on experiments result discussion were given. Finally, the summary was presented.

## 2. Methods

**Figure 1:**
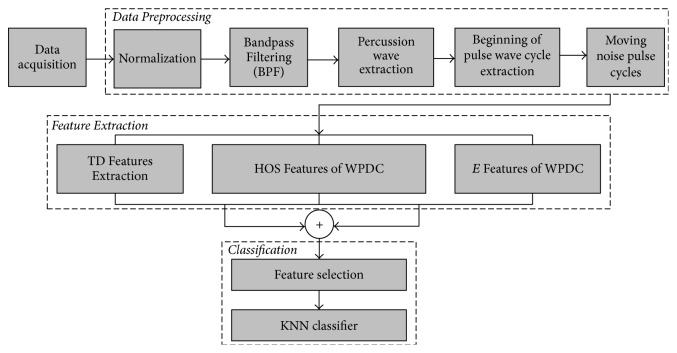
General flow diagram.

Pulse data acquisition, preprocessing, pulse wave cycle segmentation, and feature extraction and classifier evaluation were performed. The general flow diagram is shown in Figure 1.

### 2.1. Data Acquisition

Data were collected from elderly volunteer subjects who presented for physical examinations at the community health service center in Pudong New District of Shanghai. The subjects were allowed to rest for 3–5 minutes before data collection and were instructed to sit, breathe quietly, relax the upper arm, extend the forearm, and flex the shoulder and elbow to about 120°, with the left wrist on a pulse pillow. Then, our specially developed TCM pulse bracelet [14] was placed over the Guan position in the left hand to capture the best pulse signals for 10 s.

Subjects were excluded from analysis if they lacked complete data for control or outcome variables or had significant heart disease.

A total of 770 subjects met the inclusion criteria and provided 10 s of pulse data for grouping of pulse wave cycles into NG, HerG, and HestG categories, according to blood pressure values. NG subjects had a baseline systolic blood pressure < 120 mmHg or diastolic blood pressure < 80 mmHg. HerG subjects had a baseline systolic blood pressure of 130 to 139 mmHg or diastolic blood pressure 80 to 89 mmHg. HestG subjects had a baseline systolic blood pressure > 140 mmHg or diastolic blood pressure > 90 mmHg. Each group was divided into 2 classes by history of hypertension (yes or no). Pulse wave cycle data were obtained for 10 s in all subjects during data preprocessing.

### 2.2. Data Preprocessing

Baseline wandering of original pulse data was removed with a high-pass filter in the sampling device. A bandpass from 0.5 Hz to 30 Hz filter was used to smooth waves affected by tremor or breathing. A Shannon Energy Envelope, Hilbert Transform (SEEHT) extractor was used for the percussion wave and beginning of the pulse wave cycle, as it was thought to be better than other extractors for wider, small pulse waves, or sudden changes in wave amplitude [12, 15]. A pulse wave cycle was defined as the interval between two initial sets of pulse data.

**Figure 2:**
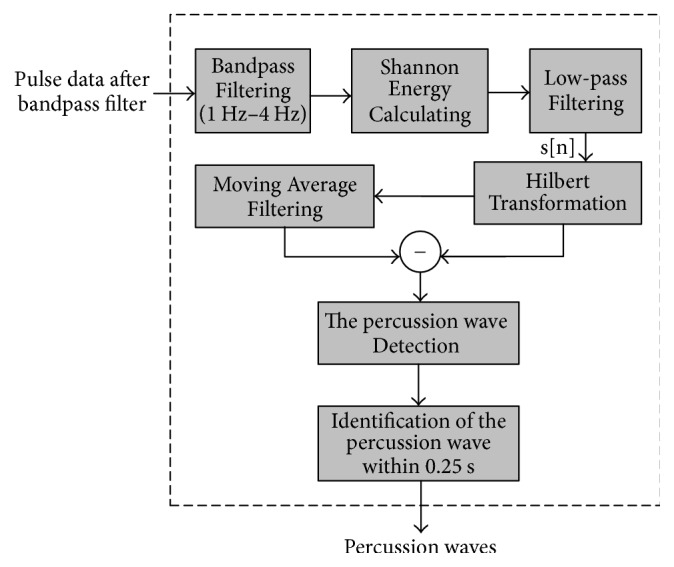
SEEHT extractor flow diagram.

SEEHT extractor for the percussion was showed in more detail in [15] (Figure 2). Firstly, a bandpass filter with 1~4 Hz is designed to exclude other peaks and emphasize the percussion wave. Secondly, the data after bandpass filter are transformed by the Shannon Energy Envelope formula. However, wrist pulse signals between 1 Hz and 4 Hz are restrained by differentiated signals. SEE signals based on differentiated signals bring abrupt changes because the other waves are amplified in differentiation. The major local maxima of smooth SEE indicate approximate locations of the percussion waves. Hence, for detecting the percussion waves, a low-pass filtering is used for smoothing SEE to reduce the complexity of searching the local maxima. Thirdly, Hilbert Transform is used for finding the peaks. And then, the moving average filter signals after Hilbert Transform, which removed the low-frequency drift, locate the peaks by zero-cross point from positive shaft to negative shaft. Finally, the real peaks of the percussion wave are the maximum within 0.25 s in the pulse data after bandpass filter with 0.5~30 Hz.

**Figure 3:**
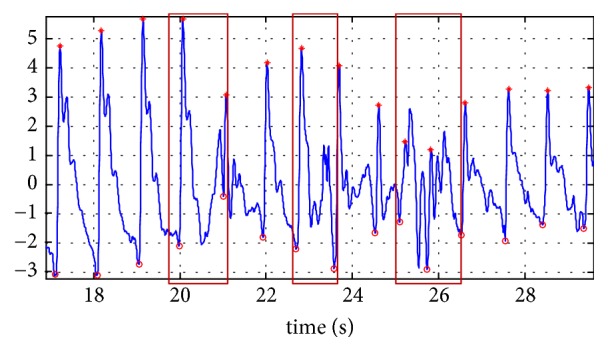
Pulse segmentation results including error parts.

Although the SEEHT method had shown good results for extraction of the percussion wave and the beginning of a pulse wave cycle, an error was observed in segmentation. This is basically due to morphological diversity in the pulse wave cycle. To eliminate the influence of segmentation error on the experimental results, noise in pulse wave cycles was excluded by visual inspection. For example, in Figure 3, there is a pulse signal (blue line) with low quality. The red asterisks are the percussion wave detected by SEEHT method. The red cycle is the start point of a pulse cycle and the end point of prior pulse cycle. So it is pulse cycle segmentation from one red cycle to next one. There are three error segmentations (red box) because of noise, so for every pulse sample segmentations result, we find out the error parts by visual inspection and delete that to ensure the effectiveness of pulse cycles in subsequent research.

### 2.3. Feature Extraction

To identify differences in pulse wave cycles between elderly hypertensive and normotensive, TD, *E*, and HOS features of WPDC were extracted after preprocessing.

#### 2.3.1. Time Domain Feature Extraction

**Figure 4:**
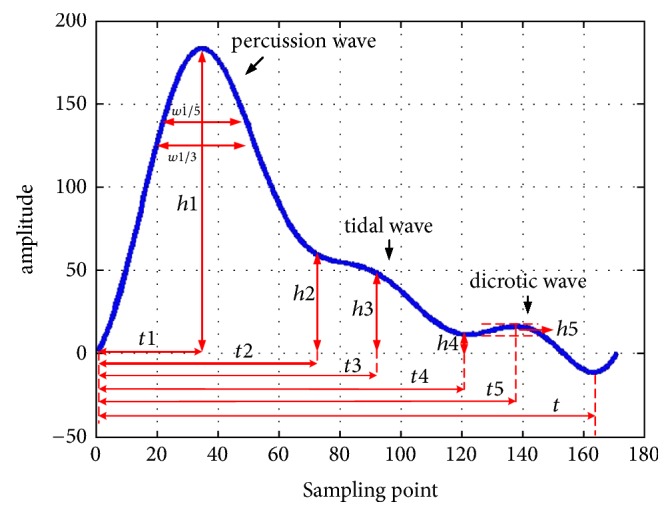
The healthy elderly pulse wave cycle.

A standard pulse wave is made up of 3 components: the percussion wave, tidal wave, and dicrotic wave. TD features include the duration and amplitude of the inflection point of 3 waves, which were extracted using a previously described method named Shap Threshold Value (STV) method (Figure 4).

**Figure 5:**
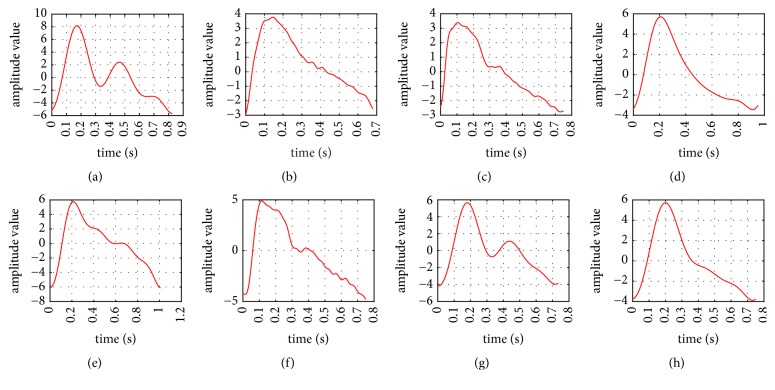
Eight pulse cycles shapes.

STV method, which was described in more detail in pages 32–35 of [12], mainly contains two steps. First is that the pulse wave cycles are classified into eight pulse cycles (in Figure 5) by the shape according to expert experience and domain knowledge. Second step is detecting the inflection point in every shape using different threshold values.

Most TD features have clear physiological significance. In this study, 20 TD features (Figure 4) were chosen for analysis including 6 duration features (*t*1, *t*2, *t*3, *t*4, *t*5, *t*), 5 amplitude features (*h*1, *h*2, *h*3, *h*4, *h*5, *h*1/*t*1, *h*3/*h*1, *h*4/*h*1), 4 width features (*w*31, *w*51, *w*31/*t*, *w*51/*t*), and 2 area features (*As, Ad*). The meaning of above features is showed in Table 1.

#### 2.3.2. Wavelet Packet Decomposition

**Figure 6:**
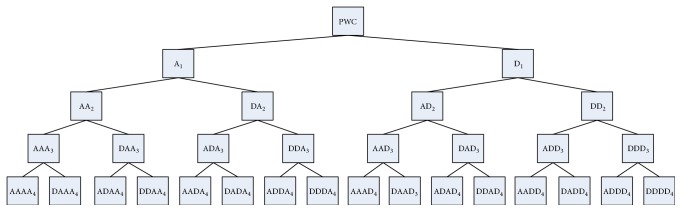
Wavelet packet analysis of pulse wave cycles with 4 levels (A: approximations, D: details; subscript (1, 2, 3, 4): levels of WPD).

The discrete wavelet transform (DWT) only decomposes low-frequency components (approximations: A). The wavelet packet method, which is an expansion of the DWT method, can increase a wide variety of skills and power of the wavelet transform [16]. Wavelet packet decomposition (WPD) utilizes both low-frequency and high-frequency components (details: D). In WPD, the approximation achieved from the first level is split into new detail and approximation components, and this process is then repeated. Mother wavelet functions are important for wavelet packet coefficients and classification accuracy of extracted features. It was reported that the best feature set was obtained with the db6 wavelet function [17]. Therefore, this study chose the db6 wavelet function as the mother wavelet function to estimate the wavelet packet coefficients. The number of decomposition levels was set at 4. Therefore, 30 subbands were obtained for the fourth level of WPD.  Figure 6 shows the fourth level of the WPD tree of pulse wave cycles.

#### 2.3.3. Higher-Order Statistics and Energy Entropy

Higher-order statistics (HOS) have been applied successfully to extract features for classification [13]. In signal processing, many signals, especially nonlinearities, cannot be examined properly by second-order statistical methods. Therefore, higher-order statistical methods have been developed. While first- and second-order statistics contain mean and variance, nonlinear combinations of higher-order moments contain cumulants [18].

Let *X*(*n*) is real, discrete time random process. The moments of *X*(*n*) are defined as the coefficients in Taylor series expansion of the moment generating function [19].

(1) $$\begin{array}{c} \phi x\left(\;w\;\right)=E\left[\;\exp\left(\;jwx\;\right)\;\right]. \end{array}$$

For zero mean discrete time signals, moments and cumulants are defined as [13]

(2) m2i=EXn,Xn+i,m3i,j=EXn,Xn+i·Xn+j,m4i,j,k=EXn,Xn+i·Xn+j·Xn+k,

where *E*[·] is defined as the expectation operation and *X*(·) is the random process.

The second characteristic function of *X*(*n*), defined as [13]

(3) $$\begin{array}{c} X\left(\;w\;\right)=\ln\phi x\left(\;w\;\right)=\ln E\left[\;\exp\left(\;jwx\;\right)\;\right], \end{array}$$

is called the cumulant generating function, and the coefficients in its Taylor expansion are the *n*th-order cumulants of *X*(*n*), represented as *c*_*n*_(*τ*_1_, *τ*_2_,…, *τ*_*n*_). The cumulants are defined as [13]

(4) c2i=m2i,c3i,j=m3i,j,c4i,j,k=m4i,j,k−m2im2j−k−m2jm2k−i−m2km2i−j.

The second-, third-, and fourth-order cumulants are calculated for each pulse cycle taking lag 0, which means that the value of *i*, *j*, *k* equals zero. The zero-lag cumulants have special names: *c*_2_(0) is the variance and is denoted by *σ*^2^; *c*3(0,0) and *c*4(0,0, 0) are denoted by *γ*3*x* and *γ*4*x* known as skewness and kurtosis, respectively.

In this study, the HOS methods are used to extract new and fewer number of features from the wavelet packet decomposition coefficients There were 30 subbands for the 4 levels as noted. Three features were extracted for each subband using HOS. We calculate HOS methods, second-, third-, and fourth-order cumulants including using cumulants functions in MATLAB 2013a:

(5) HOS_second=cum2estx,0,lengthx,0,＇biased＇,HOS_third=cum3estx,0,lengthx,0,＇biased＇,HOSfour=cum4estx,0,lengthx,0,＇biased＇.

In addition, Shannon entropy was used to calculate the energy of WPDC with the following entropy function in MATLAB 2013a:

(6) $$\begin{array}{c} E=wentropy\left(\;x,\text{＇＇shannon＇＇}\;\right), \end{array}$$

where *x* represents the wavelet packet decomposition coefficients of every pulse cycle. Thus, 30 *E* features (*E*1, *E*2 ⋯ *E*30) and 90 HOS features (HOS1, HOS2 ⋯ HOS90) were obtained for analysis.

### 2.4. Feature Selection

CfsSubsetEval and BestFirst were used for feature selection; these are built-in attribute evaluator and search methods in WEKA 3.8. CfsSubsetEval evaluates the worth of a subset of features by considering the individual predictive ability of each feature along with the degree of redundancy. Subsets of features that are highly correlated with the class while having low intercorrelation are preferred. The BestFirst method searches the space of attribute subsets by greedy hillclimbing augmented with a backtracking facility. Setting the number of consecutive nonimproving nodes allowed control of the level of backtracking. BestFirst may start with an empty set of attributes and search forward, with a full set of attributes and search backward, or at any point and search in both directions. The process is shown as Algorithm 1.

### 2.5. Classification

*k*-Nearest Neighbor (*k*-NN) [20], which is the most effective and common nonparametric method in pattern recognition classification, was used for evaluation of the effectiveness of all features. *k*-NN is independent of statistical distribution of training examples and classifies objects by computing their distance to the training examples in the feature space. The object is assigned to the class most common among its *k*-Nearest Neighbors. In this study, when *k* = 1, the object is simply assigned to the class of its nearest neighbor.

To compare the results of classification, the statistical definitions used were as follows:

- TP: true positive, number of positives;
- TN: true negative, number of negatives;
- FP: false positive, number of negatives;
- FN: false negative, number of positives;
- ROCA: receiver operating characteristic curve area, in which the *x*-axis and *y*-axis are the False Positive Rate (FPR) and True Positive Rate (TPR), respectively.

In this study, positive means hypertension history, and negative means nonhypertension history. Finally, accuracy (ACC), sensitivity (SE), specificity (SP), and ROCA ware used as evaluation indicators. The relevant formulas are shown as follows:

(7) SE=TPTP+FN×100%,SP=TNTN+FP×100%,ACC=TN+TPTP+TN=FN+FP×100%,FPR=1−SP,TPR=SE.

## 3. Experimental Results

After data preprocessing and noise removal, 6,075 pulse wave cycles were analyzed for the NG, HerG, and HestG groups, and the hypertension history and nonhypertension history classes. The 6 data sets are detailed in Table 2.

In every experiment, 20 TD features, 90 HOS features, and 30 *E* features were selected with 10-fold cross-validation. The occurrence frequency of these features was designated as OF. For example, a 5-fold selected feature was chosen 5 times in 10-fold selection (OF = 5/10 × 100 = 50%). All chosen features with different OFs were divided into different combinations by eliminating lower values. Finally, 1-NN was used to verify different feature combinations. The experimental software platform for 1-NN and feature selection was Weka 3.8. All features were extracted in MATLAB 2013a.

### 3.1. Feature Selection Results

**Figure 7:**
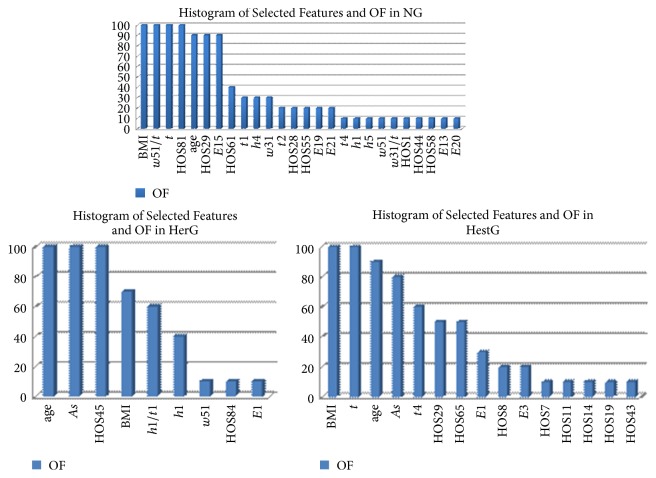
Histogram of selected features and OF in NG, HerG, and HestG.

Using 10-fold selection, 26 features were selected in experiment 1 for NG, 9 features in experiment 2 for HerG, and 15 features in experiment 3 for HestG. The selected features and OF values are shown in Table 3 and Figure 7.

### 3.2. Classification Results

All chosen features with different OF values were divided into different combinations by eliminating the lower OF in every experiment. For example, 26 features were selected in NG, including 10 with 10% OF, 5 with 20% OF, 3 with 30% OF, 1 with 40% OF, 3 with 90% OF, and 4 with 100% OF. Accordingly, NG experimental results were divided into 5 groups. The first group eliminated 10 features with 10% OF and retained the remaining features. Therefore, the last group only contained the 4 features with 100% OF. The grouping in HerG and HestG was the same as in NG. Moreover, based on all feature subgroups and all selected feature subgroups, NG, HerG, and HestG experiments were divided into 7, 6, and 9 subgroups, respectively.

**Figure 8:**
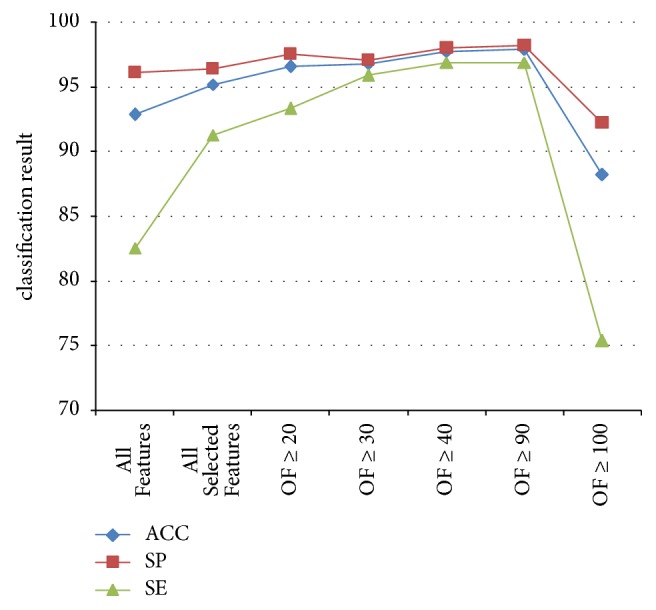
Classification results for every group in NG.

**Figure 9:**
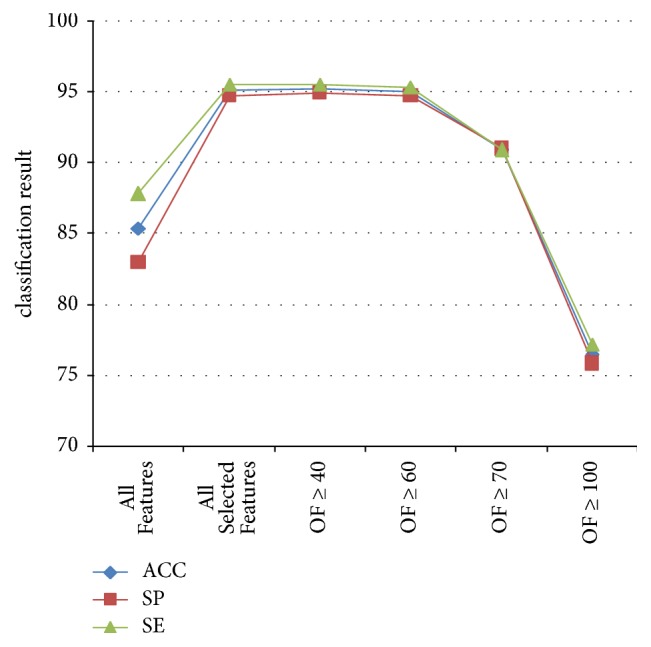
Classification results for every group in HerG.

**Figure 10:**
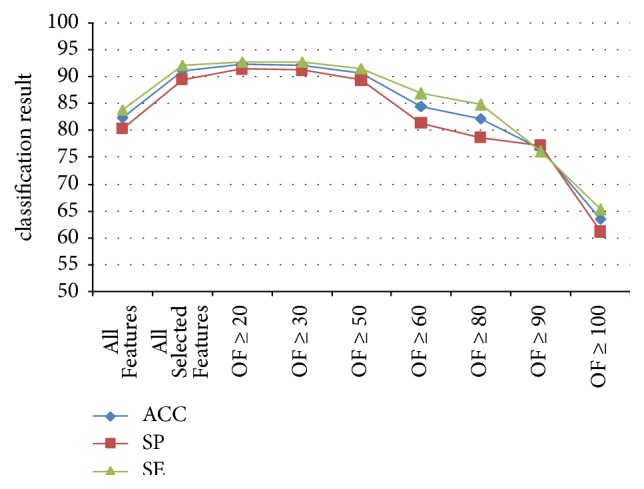
Classification results for every group in HestG.

1-NN was used in every subgroup of experiments to evaluate the effectiveness of the features groups. The classification results for NG, HerG, and HestG are shown in Tables 4, 5, and 6, respectively. To highlight the features of optimal combinations, line charts are shown in Figures 8, 9 and 10, respectively.

## 4. Discussion

In the NG experiment, 7 features were selected for best performance: age, BMI, *w*51/*t*, *t*, HOS29, HOS81, and *E*15 (Figure 8); these were found at least 9 times in 10-fold selection (OF ≥ 90). Using the same rules, the best performance features in the HerG experiment were age, BMI, *h*1, *h*1/*t*1, *As*, and HOS45, and the best performance features in the HestG experiment were age, BMI, *t*4, *As*, *t*, HOS8, HOS29, HOS65, *E*1, and *E*3.

**Figure 11:**
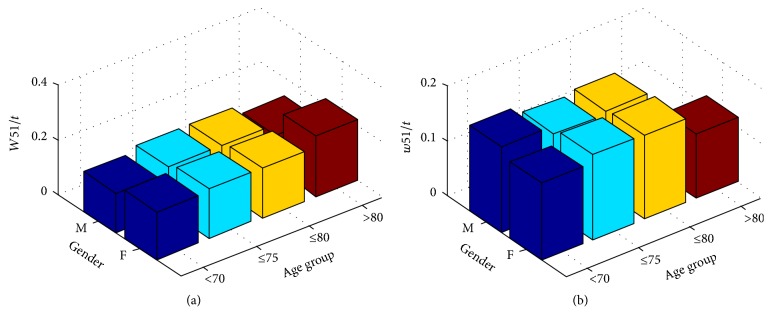
*W*51/*t* change in NG.

Age and BMI in the classification results of the 3 experiments all showed good performance, consistent with other reports. There were different trends among selected TD features between normal values in the hypertension and nonhypertension groups. For example, in the NG experiment, an increase in age was accompanied by an increase in *W*51/*t* in the group with normal blood pressure values and a history of hypertension (Figure 11(a)), but there was no consistent change in those without a history of hypertension (Figure 11(b)).

**Figure 12:**
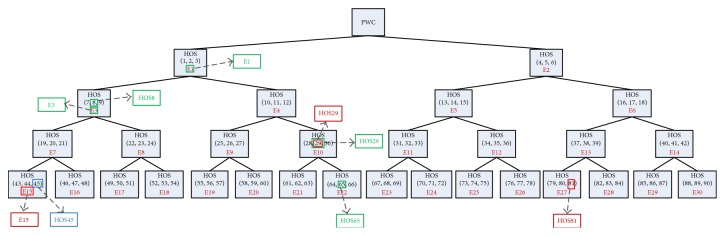
Selected HOS and *E* features of WPDC.

Most of the selected HOS and *E* features of WPDC had low-frequency components. One feature from the first level, 2 from the second level, 1 from the third level, and 4 from the fourth level (Figure 12) were selected as best features. The selected features included 3 third cumulants, 2 fourth cumulants, and 3 *E* features.

Each subband level after WPD contained second-, third-, and fourth-order cumulants. Red box denotes selected features in NG; blue box denotes selected features in HerG; green box denotes selected features in HestG.

In TCM theory, the pulse type changes from slippery to wiry with age. The consensus among TCM physicians is that hypertensive patients have a wiry pulse. Research has shown a correlation between the rank of a wiry pulse and different levels of hypertension. Two types of wiry pulse (healthy elderly wiry pulse and hypertensive wiry pulse) show a distinct difference. The classification accuracy showed a decreasing trend as blood pressure values increased (97.91% in HG, 95.24% in HerG, and 92.28% in HestG). Because of the normal blood pressure values in the NG group, there are essentially 2 classes of a wiry pulse: the healthy elderly wiry pulse and the hypertensive wiry pulse. However, in the HerG and HestG groups, the pulse wave in those without a hypertension history reflected the features of a hypertensive wiry pulse. Thus, the classification accuracy in the HerG group was lower than that in the NG group, and the accuracy in the HestG group was lower than that in the HerG group. The features selected in the classification all achieved accuracy of greater than 92.28% in the 3 groups. Although the features of a hypertensive wiry pulse were present in 2 classes (hypertension and nonhypertension history) in the HerG and HestG groups, the selected features can also reflect cardiovascular function under conditions of sustained hypertension.

## 5. Conclusion

In elderly individuals, pulse wave cycle features in the same blood pressure range show significant differences according to hypertension history. Recognition rates of over 90% have been achieved in classification experiments using the selected features. This shows that not all equivalent blood pressure levels represent the same cardiovascular function. Meanwhile, the TD, HOS, and energy features of WPDC can be used to evaluate cardiovascular function according to blood pressure values.

This study shows that management of health risk requires more than blood pressure medication in elderly individuals with hypertension. Changes in pulse wave and blood pressure values should be used in an evaluation index. Future research will focus on finding more effective features for assessment of blood vessels and analysis of the relationship between pulse features and central arterial pressure.

## Figures and Tables

**Algorithm 1:**
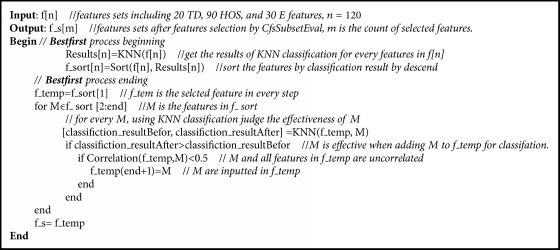
CfsSubsetEval and BestFirst.

**Table 1:** TD features.

Id	TD features	Meaning
1	*t*1	The duration of the percussion wave
2	*t*2	The duration of the beginning of the tidal wave
3	*t*3	The duration of the crest of the tidal wave
4	*t*4	The duration of the beginning of the dicrotic wave
5	*t*5	The duration of the crest of the dicrotic wave
6	*h*1	The amplitude of the percussion wave
7	*h*2	The amplitude of the beginning of the tidal wave
8	*h*3	The amplitude of the crest of the tidal wave
9	*h*4	The amplitude of the beginning of the dicrotic wave
10	*h*5	The amplitude of the crest of the dicrotic wave
11	*w*31	The width in 1/3 amplitude of the percussion wave
12	*w*51	The width in 1/5 amplitude of the percussion wave
13	*w*31/*t*	The ratio of *w*31 and *t*
14	*w*51/*t*	The ratio of *w*51 and *t*
15	*h*1/*t*1	The ratio of *h*1 and *t*1
16	*h*3/*h*1	The ratio of *h*3 and *h*1
17	*h*4/*h*1	The ratio of *h*4 and *h*1
18	*t*	A pulse cycle duration
19	*As*	The systolic pulse wave area in *t*4
20	*Ad*	The diastolic pulse wave area between the end of *t*4 and the end of *t*

**Table 2:** Pulse wave cycle distribution.

Blood pressure	Hypertension history	Pulse wave cycle count	Age (mean ± std)	Male/female
Normal (NG)	No	619	72 ± 5	1 : 1.63 (235 : 384)
	Yes	195	73 ± 5	1.24 : 1 (108 : 87)

Higher (HerG)	No	534	71 ± 6	1 : 1.34 (228 : 306)
	Yes	517	71 ± 6	1.01 : 1 (259 : 257)

Highest (HestG)	No	1779	72 ± 6	1 : 1.46 (724 : 1055)
	Yes	2431	72 ± 6	1 : 1.62 (927 : 1504)

*Total*		*6075*	*72 ± 6*	*1 : 1.45* *(2,481 : 3,594)*

**Table 3:** Selected features in NG, HerG, and HestG groups.

Experiment group	ID	Feature	OF (%)
Experiment 1 NG	1	Age	90
	2	BMI	100
	3	*t*1	30
	4	*t*2	20
	5	*t*4	10
	6	*h*1	10
	7	*h*4	30
	8	*h*5	10
	9	*w*31	30
	10	*w*51	10
	11	*w*31_*t*	10
	12	*w*51_*t*	100
	13	*t*	100
	14	HOS1	10
	15	HOS28	20
	16	HOS29	90
	17	HOS44	10
	18	HOS55	20
	19	HOS58	10
	20	HOS61	40
	21	HOS81	100
	22	*E*13	10
	23	*E*15	90
	24	*E*19	20
	25	*E*20	10
	26	*E*21	20

Experiment 2 HerG	1	age	100
	2	BMI	70
	3	*h*1	40
	4	*w*51	10
	5	*h*1_*t*1	60
	6	*As*	100
	7	HOS45	100
	8	HOS84	10
	9	*E*1	10

Experiment 3 HestG	1	age	90
	2	BMI	100
	3	*t*4	60
	4	*As*	80
	5	*t*	100
	6	HOS7	10
	7	HOS8	20
	8	HOS11	10
	9	HOS14	10
	10	HOS19	10
	11	HOS29	50
	12	HOS43	10
	13	HOS65	50
	14	*E*1	30
	15	*E*3	20

**Table 4:** Classification results for every group in NG.

	All features	All selected features	OF ≥ 20	OF ≥ 30	OF ≥ 40	OF ≥ 90	OF ≥ 100
ACC	92.87	95.21	96.56	96.81	97.79	97.91	88.21
SP	96.12	96.45	97.58	97.09	98.06	98.22	92.25
SE	82.56	91.28	93.33	95.9	96.92	96.92	75.38
ROCA	0.892	0.933	0.957	0.966	0.976	0.974	0.841

**Table 5:** Classification results for every group in HerG.

	All features	All selected features	OF ≥ 40	OF ≥ 60	OF ≥ 70	OF ≥ 100
ACC	85.34	95.15	95.24	95.05	90.96	76.5
SP	82.96	94.76	94.94	94.76	91.01	75.84
SE	87.81	95.55	95.55	95.36	90.91	77.18
ROCA	0.852	0.95	0.951	0.947	0.912	0.758

**Table 6:** Classification results for every group in HestG.

	All features	All selected features	OF ≥ 20	OF ≥ 30	OF ≥ 50	OF ≥ 60	OF ≥ 80	OF ≥ 90	OF ≥ 100
ACC	82.38	91.07	92.28	92.14	90.64	84.56	82.23	76.56	63.52
SP	80.27	89.49	91.46	91.23	89.38	81.39	78.7	77.23	61.1
SE	83.92	92.23	92.88	92.8	91.57	86.88	84.82	76.06	65.28
ROCA	0.821	0.909	0.922	0.92	0.905	0.842	0.818	0.814	0.667

## References

[1] Velik R. (2015). An objective review of the technological developments for radial pulse diagnosis in Traditional Chinese Medicine. *European Journal of Integrative Medicine*.

[2] Zhao C., Li G.-Z., Wang C., Niu J. (2015). Advances in patient classification for traditional chinese medicine: a machine learning perspective. *Evidence-Based Complementary and Alternative Medicine*.

[3] Wang N. Y., Yu Y. H., Huang D. (2015). Pulse signals analysis of fatty liver disease and cirrhosis patients by using machine learning. *The Scientific World Journal*.

[4] Nambi V., Chambless L., Folsom A. R. (2010). Carotid intima-media thickness and presence or absence of plaque improves prediction of coronary heart disease risk: the ARIC (Atherosclerosis Risk In Communities) study. *Journal of the American College of Cardiology*.

[5] Fei Z. F. (2013). *Contemporary Sphygmology in Traditional Chinese Medicine*.

[6] Ribeiro de Moura N. G., Cordovil I., de Sá Ferreira A. (2016). Traditional Chinese medicine wrist pulse-taking is associated with pulse waveform analysis and hemodynamics in hypertension. *Journal of Integrative Medicine*.

[7] Ribeiro de Moura N. G., de Sá Ferreira A. (2016). Pulse waveform analysis of chinese pulse images and its association with disability in hypertension. *JAMS Journal of Acupuncture and Meridian Studies*.

[8] Cogswell R., Pritzker M., De Marco T. (2014). Performance of the REVEAL pulmonary arterial hypertension prediction model using non-invasive and routinely measured parameters. *The Journal of Heart and Lung Transplantation*.

[9] Lee B. J., Jeon Y. J., Ku B., Kim J. U., Bae J.-H., Kim J. Y. (2015). Association of hypertension with physical factors of wrist pulse waves using a computational approach: A pilot study. *BMC Complementary and Alternative Medicine*.

[10] Shang Q. Q., Wang Y. Q. (2016). Thought and prospect on evaluation of vascular function of hypertension patients based on pulse manifestation analysis. *Journal of Traditional Chinese Medicine and Pharmacy*.

[11] Paynter N. P., Cook N. R., Everett B. M., Sesso H. D., Buring J. E., Ridker P. M. (2009). Prediction of Incident Hypertension Risk in Women with Currently Normal Blood Pressure. *American Journal of Medicine*.

[12] Hu X. J. (2013). *signal perception and computer aided recognition of traditional Chinese medicine pulse diagnosis [Ph.D. thesis]*.

[13] Yakup K., Damla K. (2012). Feature extraction for ECG heartbeats using higher order statistics of WPD coefficients. *Computer Methods and Programs in Biomedicine*.

[14] Xu J. T., Cui J., Tu L. P. (2016). *A Bluetooth Bracelet of Pulse and Pulse Data Transmission Method, China Patent, Patent No: 201610298414.6*.

[15] Hu X., Zhu H., Xu J., Xu D., Dong J. Wrist pulse signals analysis based on deep convolutional neural networks.

[16] Misiti M., Misiti Y., Oppenheim G., Poggi J. M. (2004). Wavelet toolbox for use with Matlab, User’s Guide, Ver. 3, The Math Works, Inc. *Users Guide*.

[17] Kutlu Y., Kuntalp D. (2010). *Multi-Stage Classification of Abnormal Patterns in EEG And ECG Using Model-Free Methods [Ph.D. thesis]*.

[18] Mendel J. M. (1991). Tutorial on higher-order statistics (spectra) in signal processing and system theory: theoretical results and some applications. *Proceedings of the IEEE*.

[19] Yu S., Chen Y. (2009). Noise-tolerant electrocardiogram beat classification based on higher order statistics of subband components. *Artificial Intelligence in Medicine*.

[20] Cover T. M., Hart P. E. (1967). Nearest neighbor pattern classification. *IEEE Transactions on Information Theory*.

